# Influence of the Antithrombotic Therapy in the Healing of Simple Post-Extraction Sockets: A Randomized Clinical Trial

**DOI:** 10.3390/jcm11133654

**Published:** 2022-06-24

**Authors:** Gilberto Sammartino, Roberta Gasparro, Gianrico Spagnuolo, Alessandra Miniello, Andrea Blasi, Gaetano Marenzi

**Affiliations:** Department of Neurosciences, Reproductive and Odontostomatological Sciences, University of Naples “Federico II”, 80131 Naples, Italy; gilberto.sammartino@unina.it (G.S.); roberta.gasparro@unina.it (R.G.); gspagnuo@unina.it (G.S.); andreablasi79@gmail.com (A.B.); gaetano.marenzi@unina.it (G.M.)

**Keywords:** would healing, antiplatelet, dental extraction

## Abstract

Background: An adequate blood supply plays a leading role in the healing process of the post-extractive socket; its coagulation leads to fibrin clot formation, which acts as a physical barrier able to prevent postoperative bleeding and microbial infection. The purpose of this study was to evaluate the effectiveness of antiaggregant drugs in healing post-extraction sockets compared to natural wound healing. Methods: This was a single-center prospective clinical trial. Extraction sockets allocated in healthy patients and in patients assuming antiplatelet drugs were considered. Thirty consecutive patients under (treated with/in treatment with) oral antiplatelet treatment were enrolled in the test group. In order to provide a control group, 30 consecutive patients meeting all the exclusion and inclusion criteria were enrolled. The extraction of the mono-radicular tooth was atraumatically performed without gingivoplasty or osteotomy procedures that could influence the healing process. Photographs were obtained before and immediately after surgery and at 3-, 7-, 14- and 28-days follow-up. Results: All patients assumed the prescribed therapy and their post-operative recovery was uneventful without any kind of post-extractive complications. The results of inter-group comparison show that on the third and seventh days of follow-up, the antiplatelet group expressed a statistically significant higher level of healing compared to the control group (*p* < 0.05), while no statistically significant differences were recorded at 14- and 28-days follow-up. Conclusions: Patients treated with antiplatelet agents seemed to show that this therapy can positively affect the healing process after tooth extractions.

## 1. Introduction

The most commonly performed procedure in oral surgery is tooth extraction, which follows a multifactorial healing process and can be influenced by many clinical conditions. Normal healing of the extraction site is dependent on blood clot formation, its progression to an organized matrix, and the formation of bone. An adequate blood supply plays a leading role in the healing process of the post-extractive socket; its coagulation leads to a fibrin clot formation, which acts as a physical barrier able to prevent postoperative bleeding and microbial infection [1,2,3].

Systemic comorbidities (diabetes, hypertension, chronic obstructive pulmonary disease, anemia, malnutrition, hepatic and kidney disease, etc.) are related to an impaired angiogenesis response, a decreased collagen synthesis and a reduced oxygen supply, which can negatively influence the healing of the extraction wound, reducing stable clot formation. Habits such as smoking have been highlighted as the cause of a delay in healing of the post extractive site. In vitro studies have described how nicotine negatively affects osteoblasts and inhibits the growth of fibroblasts and the production of collagen and fibronectin, while promoting collagen breakdown [4].

Drug consumption (immuno-suppression and prolonged corticosteroid therapy) is related to intra- and/or post-operative complications during post-extractive wound healing. Anticoagulant and antiplatelet drugs, usually prescribed to treat many pathologies (cardiac disease, deep venous thrombosis, pulmonary embolism, atrial fibrillation, cardiac valvular disease, prosthetic cardiac valves, cerebral accident, etc.) modify the hemostatic efficacy and the blood perfusion of the post extraction socket [5]. Their suspension to prevent intra-operative and post-operative complications of dental extractions is controversial. The risk of thromboembolic events has been documented in patients who suspended the medical treatment. Many studies considering dental extractions as surgical treatment with no severe bleeding complications have suggested avoiding therapy suspension or modification. Current guidelines suggest that for low-risk procedures, modern anticoagulant and antiplatelet drugs are considered to be safe and extractions can be performed without interrupting or altering therapy [6,7,8]. However, there is no evidence regarding the ability of these drugs to influence blood supply and stable clot formation in the post-extraction site. The purpose of this study was to evaluate the effectiveness of the antiaggregant drugs on the healing of post-extraction sockets compared to natural wound healing. The authors hypothesized that this therapy could improve the healing of the post extraction socket.

## 2. Materials and Methods

This was a single-center prospective clinical trial. Extraction sockets allocated in healthy patients and patients assuming antiplatelet drugs were considered. All patients possessing the inclusion criteria were eligible to be admitted to this study, with neither ethnic nor gender differentiation. Ethical approval was obtained by the Health Sciences Human Ethics Sub-Committee of Federico II (Prot. No. 370 of 9 December 2020). The clinical study was registered on ClinicalTrial.gov registry (registration number NCT0433744) and the present trial was conducted according to the CONSORT statement (http://www.consort-statement.org/ (accessed on 25 July 2019)).

Study population

From December 2020 to December 2021, the cohort of patients belonging to the University Federico II of Naples (Italy) were considered eligible for admission to the study in accordance with the inclusion criteria:

- Patients above 18 years;
- Patients following long-term intake of oral anti-platelet therapy;
- Patients requiring extractions of a minimum of 1 single-rooted tooth/root.

The demographic characteristics of the ruled patients are summarized in Table 1.

Detailed information regarding the procedures were received by the patients and an informed consent form was signed by each patient admitted to the study. Each Patient was adequately informed regarding the aspects of the study, potential risks and treatment alternatives.

All patients were subjected to anamnestic data collection, including demographic and clinical variables (gender, age, comorbidities), voluptuous habits (smoking and alcohol consumption) and antiplatelet therapy (drug and dose).

All patients underwent blood tests to evaluate the hematocrit and the platelet number, with a minimum of one professional oral hygiene session prior to the performance of the dental extractions. In addition to requesting a pre-operative radiograph (intraoral film or panoramic film), follow-ups were performed, firstly from immediate post extraction and then 2 weeks after.

Exclusion criteria were:

- Patients refusing participation in the study and/or without written informed consent;
- Patients possessing a history of hypersensitivity or local anesthetic allergies;
- Patients following a treatment protocol of oral antiplatelet therapy for a period of less than three months;
- Immuno-supressed and immuno-compromised patients;
- Disabled patients;
- Patients with a state of acute infection related to the tooth to be extracted;
- Patients in treatment or treated with amino-bisphosphonates or antiresorptive drugs;
- Psychiatric patients;
- Patients with thrombocytopenia: primary or secondary to another disease (platelets < 40,000);
- Essential coagulation disorders patients (e.g., thrombocytosis);
- Congenital coagulation deficiencies patients (e.g., hemophilia);
- Patients with severe cirrhosis;
- Patients with severe chronic kidney disease;
- Patients with uncompensated diabetes;
- Patients who are pregnant or nursing;
- Patients requiring socket preservation in any of the included extractions sites;
- Patients performing radio- therapy and/or chemotherapy.

Thirty consecutive patients under oral antiplatelet treatment were enrolled in test group (Group A). In order to provide a control group (Group B), 30 consecutive patients meeting all the exclusion and inclusion (excluding the oral antiplatelet therapy) criteria were enrolled.

**Figure 1:**
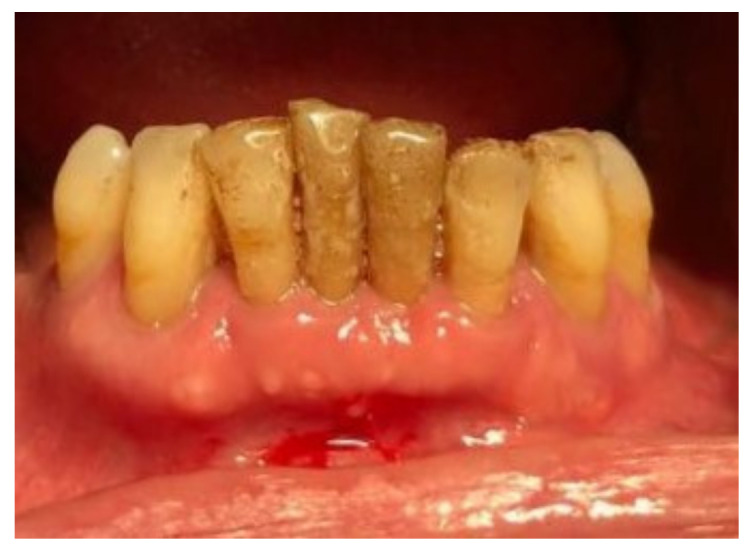
Pre-surgical condition, Group A.

**Figure 2:**
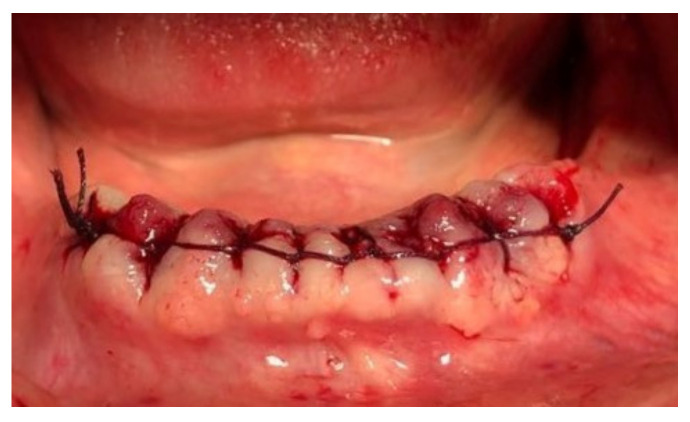
Immediate post-surgical condition, Group A.

**Figure 3:**
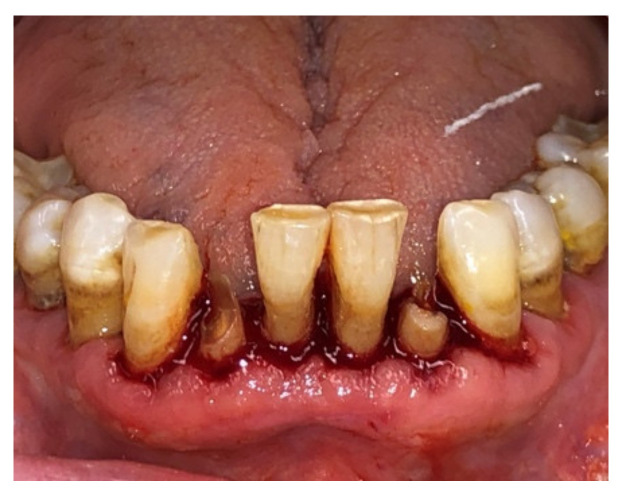
Pre-surgical condition, Group B.

**Figure 4:**
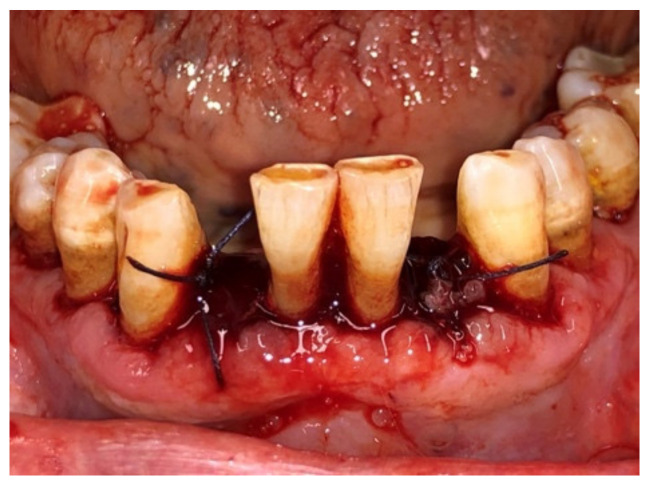
Immediate post-surgical, Group B.

Before surgery, all patients underwent blood tests to evaluate the blood count, the hepatic and kidney functions and an oral hygiene session. All patients also had 2 g of amoxicillin (because of their vulnerability) and were instructed not to suspend the antiplatelet therapy. After infiltration of local anesthesia (mepivacaine 3%), the extraction of the mono-radiculated tooth was atraumatically performed without gingivoplasty or osteotomy procedures that could influence the healing process (Figure 1, Figure 2, Figure 3 and Figure 4).

**Figure 5:**
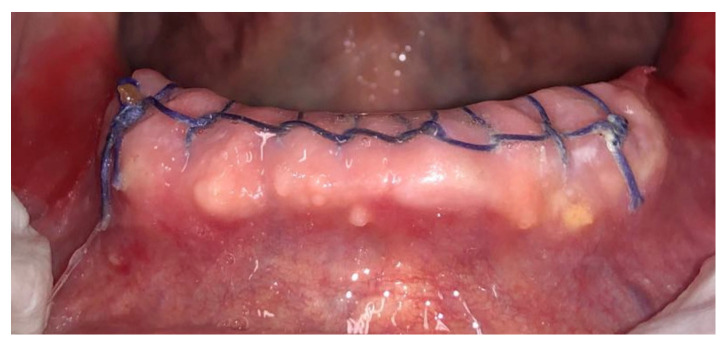
3 days after surgery, Group A.

**Figure 6:**
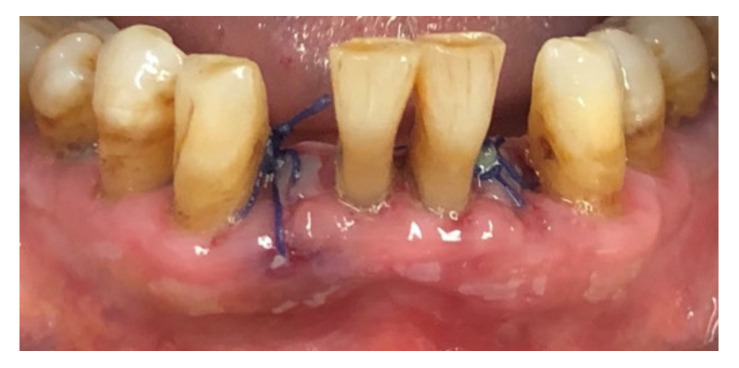
3 day after surgery, Group B.

**Figure 7:**
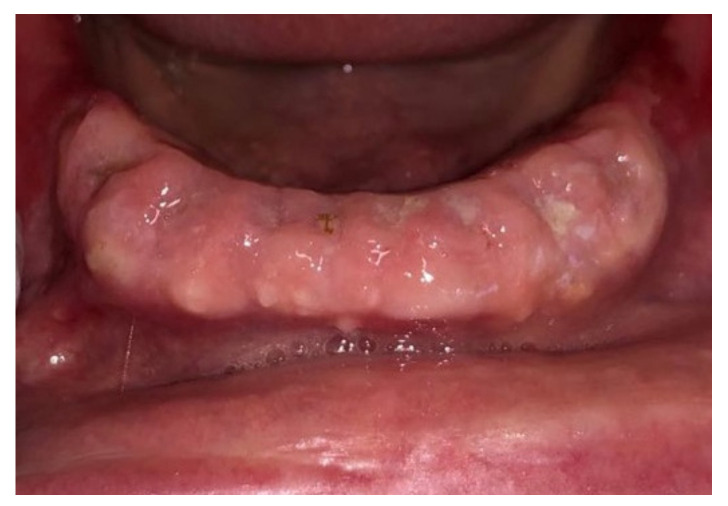
7 days after surgery, Group A.

**Figure 8:**
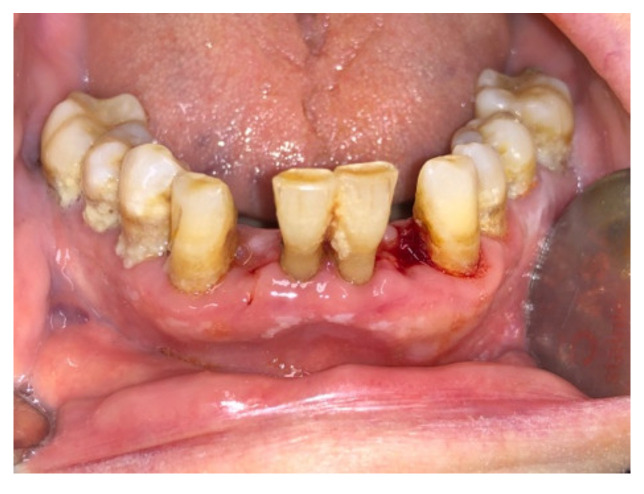
7 days after surgery, Group B.

After tooth extraction, all the sockets were carefully curetted. All sites were sutured with resorbable sutures. Post-surgery therapy was prescribed: antibiotic (amoxycillin and clavulanic acid each 12 h for 5 days), mouthwash with chlorhexidine 0.12% and paracetamol in case of pain. Sutures were removed after 7 days. Photographs were obtained for each group, before and immediately after surgery (Figure 1, Figure 2, Figure 3 and Figure 4) and at 3 days (Figure 5 and Figure 6), 7 days (At the time of sutures removal) (Figure 7 and Figure 8), 2 weeks and 4 weeks follow-up.

The provided pictures were examined by three clinicians in order to provide a post-extractive healing evaluation of the site. The clinicians were unaware of the reference group (single blind). Each wound was scored a rate from 1 to 5 (very poor, poor, good, very good, excellent), following the Landry et al. index [9].

Evaluations of tissue color, granulation tissue presence, incision margins evaluation (epithelialization and exposure of the connective tissue) and the presence or absence of suppuration (Table 2) were included in the index.

### Statistical Analysis

Scores based on Landry’s Wound Healing Index assigned to post-extraction sockets in both the test and control group were recorded at 3-, 7-, 14- and 28 days follow-up.

A Shapiro–Wilk test was performed to assess the normal distribution of data. Data were reported as means and standard deviations and medians and inter-quartile ranges (IQR).

Since the data were not normally distributed, a comparison between groups was performed at each time point with a non-parametric test (Mann–Whitney test).

An intra-group comparison, between scores recorded at each time point, was performed both for the healthy and antiaggregant group using the Friedman test.

A *p*-value < 0.05 was accepted as statistically significant.

All analyses were performed with statistical software (IBM SPSS Statistics for Windows, Version 25.0. IBM Corp., Armonk, NY, USA)

## 3. Results

Sixty patients participated to the study, 30 proceeding from the test group (Group A) (antiplatelet therapy) and 30 from the control group (Group B). All patients consumed the prescribed therapy and no post-extractive complications or any kind of events were observed during postoperative recovery. All patients were present at follow-up recalls.

Table 3 shows the Wound Healing Scores for both test and control groups expressed as medians and IQRs, as well as means and standard deviations. As expected, in each group, the values increased from the third to the twenty-eighth day, thus indicating the progressive healing of the site. The intra-group analysis showed a statistically significant (*p* < 0.05) increase in healing index over time for both the test and control group.

The results of inter-group comparison show that on 3 and 7 day follow-up, the antiplatelet group expressed a statistically significant higher level of healing compared to the control group (*p* < 0.05), while no statistically significant differences were recorded at 14 and 28 days follow-up.

**Figure 9:**
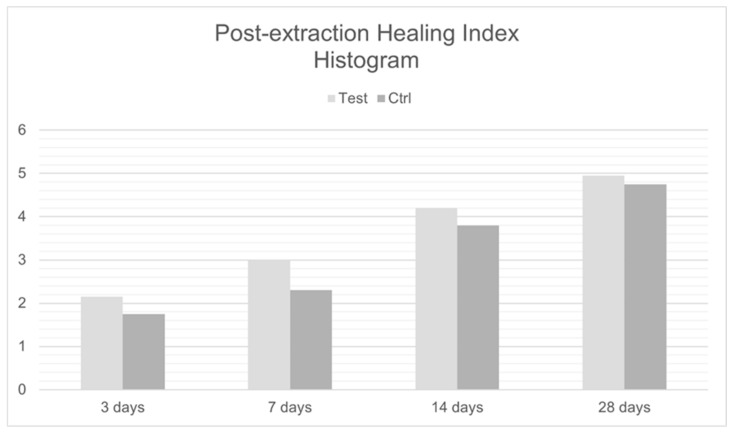
Post-extraction Healing Index Histogram.

**Figure 10:**
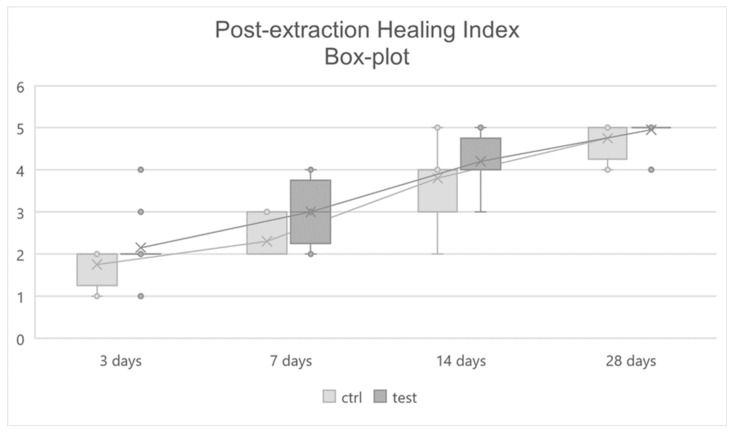
Post-extraction Healing Index Box-plot.

Data recorded by means and standard deviations and by medians and IQRs are also graphically shown in (Figure 9) and (Figure 10), respectively.

## 4. Discussion

The management of post-extraction bleeding in patients undergoing anticoagulant or antiplatelet therapy has always been a controversial point for oral surgeons.

Halley et al., in a review that included three randomized controlled trials and seven controlled trials, mentioned that the risk of postoperative bleeding was significantly higher in patients on anti-aggregation therapy, but the bleeding time was not significantly different between cases and controls [10].

The results of a meta-analysis by Zhao et al. conducted on randomized control trials (RCTs) and non-randomized control trials showed that the risk of postoperative bleeding was significantly higher in patients taking Aspirin, but that bleeding time was not significantly different in the two groups [11].

In a case–control study by Sadeghi-Ghahrody et al. conducted on 64 patients during the first year after coronary stent insertion on aspirin and clopidogrel therapy and 50 healthy patients engaged as controls, there were no significant differences in terms of bleeding assessed 30 min after surgery. During the 48-hrs following the surgery, there were no uncontrolled bleeding or emergency reports [12].

These studies show how the dental extraction procedure in cardiopathic patients can procure a risk of thromboembolic events. The antiplatelet drugs must therefore not be suspended for dental extractions or minor surgical interventions because the risk of bleeding is not significant and can be effectively controlled through local hemostasis. Adopting the most current guidelines, it was therefore decided not to suspend the drug before surgery.

In the post extractive phase, the alveolus, thanks to an adequate blood supply, is occupied by the clot that allows the onset of healing phenomena.

The formation of the clot leads to the birth of a fibrin matrix, in which platelets are immersed together with small quantities of plasma fibronectin, vitronectin and thrombospondin. The clot plays the role of a physical barrier against the microbial infection; it is a reservoir of growth factors and cytokines, thus providing a favorable environment for cell migration. Neutrophils arrive at the wound site within minutes of injury, succeeded by monocytes and lymphocytes. The following phase begins with migration and proliferation of the keratinocytes, then by that of the fibroblasts. Angiogenesis and axonal growth occur later. After some days, the remodeling and re-epithelialization phase occurs. The whole process is regulated by various growth factors and cytokines [13,14,15,16]. Therefore, many cell types, including blood cells, fibroblasts and endothelial cells, participate in the final healing process, and each cell type can specifically affect the function of other cell types through cell-to-cell contacts, so that the cell matrix produces and releases soluble factors [17].

Functional and structural support for the cells and tissues are provided by the extra cellular matrix, consisting of several molecules such as heparin sulfate, collagen, fibronectin, proteoglycans, chondroitin sulfate, elastin, hyaluronic acid and laminin. Some plasma proteins, including fibronectin, fibrin and thrombospondin, play a role as a temporary ECM (extracellular matrix). Soluble factors include nucleotides, cytokines, hormones, chemokines, electrolytes, free ions and growth factors [18].

Normal wound repair includes a vigorous angiogenic response that delivers nutrients and inflammatory cells to the damaged tissue [19].

Wound angiogenesis mediators include multiple soluble factors which have been identified in many wound healing models [20,21].

In light of these assessments, the fundamentality of adequate blood supply in the healing process of a wound is clear.

Therefore, patients with concomitant pathologies that compromise the healing process, such as diabetes, which causes a reduction in peripheral micro-circulation, and patients consuming bisphosphonates, were excluded from the study, because these drugs accumulate in the jawbone and/or maxilla are toxic to bone tissue and can cause soft tissue injury healing failure [22] and hemopathies. Immunosuppressed patients or patients on corticosteroid therapy, which inhibit the synthesis of cytokines essential for healing, subjects undergoing radiotherapy or chemotherapy, which increases healing times by reducing the number of monocytes available, or suffering from HBV, HCV and HIV, were also excluded.

In the light of these assessments, the fundamental role that an adequate blood supply plays in the healing process of a wound is clear. Moreover, on the 3rd and 7th day, patients undergoing antiplatelet therapy demonstrated better recovery than the other two groups of patients. It is hypothesized that this result in patients undergoing antiplatelet therapy is due to the greater blood supply at the wound level and to the longer bleeding time with a consequent increase in the site of the cells necessary for healing. Randomized control studies with a large sample size are needed in the future to confirm the data from this pilot study. Histological investigations of the healing site could provide further details in evaluating the different healing of post-extraction sites in patients taking anticoagulants or antiplatelet agents.

## 5. Conclusions

Patients treated with antiplatelet agents seemed to demonstrate that this therapy can positively affect the healing processes after tooth extractions. This could be attributed to the increased blood supply involved in the healing process. With an elevation in the number of cases encountered, if such trends are to be confirmed, the utilization of antiplatelet agents might be adopted in patients with metabolic problems and delays in wound healing, or in patients with impaired osteomucosal metabolism to improve regenerative processes, or to help to prevent osteonecrotic processes.

## Figures and Tables

**Table 1:** Demographic data of patients.

NAME	AGE	GENDER	GROUP
F. A.	53	male	Control
D. C.	48	male	Control
N. L.	69	female	Control
B. T.	22	female	Control
C. V.	65	male	Control
C. S.	50	male	Control
D. A.	43	male	Control
C. M.	56	male	Control
B. C.	47	male	Control
C. E.	51	female	Control
B. G.	55	male	Control
R. A.	61	female	Control
P. C.	56	female	Control
T. A.	47	female	Control
E. A.	50	female	Control
L. M.	46	female	Control
I. L.	68	male	Control
M. M.	60	male	Control
I. A.	54	male	Control
C. M.	48	female	Control
D. M.	64	male	Control
E. M.	77	male	Control
C. A.	64	female	Control
G. A.	61	female	Control
A. V.	63	female	Control
I. G.	78	male	Control
M. R.	54	female	Control
P. M.	60	female	Control
S. A.	63	male	Control
P. S.	43	female	Test
D. T.	78	female	Test
P. P.	89	female	Test
D. M.	45	female	Test
A. R.	74	male	Test
L. A.	65	male	Test
L. L.	77	male	Test
M. B.	71	female	Test
R. A.	55	female	Test
B. S.	76	male	Test
L. G.	50	male	Test
A. F.	64	male	Test
A. V.	72	male	Test
B. A.	58	male	Test
D. R.	60	male	Test
I. A.	67	female	Test
P. S.	73	male	Test
F. G.	56	male	Test
P. R.	64	male	Test
M. M.	70	female	Test
A. M.	60	male	Test
C. B.	26	female	Test
E. C.	73	male	Test
M. F.	59	male	Test
S. T.	63	male	Test
M. F.	87	female	Test
C. R.	50	male	Test
G. T.	67	male	Test
O.M.	48	male	Test

**Table 2:** Landry et al. [9] index.

Very Poor	Tissue colour:	50% of gingiva red
	Granulation tissue:	Present
	Incision margin:	Not epithelialized, with loss of epithelium beyond incision margin
	Suppuration:	Present
2. Poor	Tissue colour:	50% of gingiva red
	Granulation tissue:	Present
	Incision margin:	Not epithelialized, with connective tissue exposed
	Suppuration:	None
3. Good	Tissue colour:	50% of gingiva red
	Granulation tissue:	None
	Incision margin:	No connective tissue exposed
	Suppuration:	None
4. Very Good	Tissue colour:	25% of gingiva red
	Granulation tissue:	None
	Incision margin:	No connective tissue exposed
	Suppuration:	None
5. Excellent	Tissue colour:	All tissues pink
	Granulation tissue:	None
	Incision margin:	No connective tissue exposed
	Suppuration:	None

**Table 3:** Wound Healing Scores for both test and control groups expressed as medians and IQRs, as well as means and standard deviations.

	Test Group		Control Group		Significance
	Mean ± Std Dev	Median; IQR	Mean ± Std Dev	Median; IQR	
3 days	2.15 ± 0.67	2; 0	1.75 ± 0.44	2; 0.25	0.038
7 days	3 ± 0.73	3; 0.5	2.3 ± 0.47	2; 1	0.002
14 days	4.2 ± 0.52	4; 0.25	3.8 ± 0.77	4; 1	0.074
28 days	4.95 ± 0.22	5; 0	4.75 ± 0.44	5; 0.25	0.080
Significance	0.0001		0.0001		
